# Assessment of Mutational Load in Biopsy Tissue Provides Additional Information About Genomic Instability to Histological Classifications of Barrett's Esophagus

**DOI:** 10.1007/s12029-013-9570-y

**Published:** 2014-01-09

**Authors:** Harshit S. Khara, Sara A. Jackson, Saraswathi Nair, Georgios Deftereos, Shweta Patel, Jan F. Silverman, Eric Ellsworth, Cameron Sumner, Brendan Corcoran, Dennis M. Smith, Sydney Finkelstein, Seth A. Gross

**Affiliations:** 1 Norwalk Hospital, Norwalk, CT USA; 2 RedPath Integrated Pathology, Pittsburgh, PA USA; 3 Allegheny General Hospital, West Penn Allegheny Health System, Pittsburgh, PA USA; 4 NYU Langone Medical Center, New York, NY USA; 5 RedPath Integrated Pathology, Inc., 2515 Liberty Avenue, Pittsburgh, PA 15222 USA

**Keywords:** Mutational analysis, Barrett's esophagus, Esophageal adenocarcinoma, Loss of heterozygosity, Microsatellite instability

## Abstract

**Purpose:**

Progression of Barrett's esophagus (BE) to esophageal adenocarcinoma (EAC) is associated with accumulated genomic instability. Current risk stratification of BE for EAC relies on histological classification and grade of dysplasia. However, histology alone cannot assess the risk of patients with inconsistent or non-dysplastic BE histology. We, therefore, examined the presence and extent of genomic instability in advanced and less advanced BE histology using mutational load (ML).

**Methods:**

ML summarized the presence and clonality of loss of heterozygosity (LOH) mutations and the emergence of new alleles, manifested as microsatellite instability (MSI) mutations, in ten genomic loci around tumor suppressor genes associated with EAC. The ML of 877 microdissected targets from BE biopsies was correlated to their histology. Histological targets were categorized into three levels: no ML, low ML, and high ML.

**Results:**

Increasing ML correlated with increasingly severe histology. By contrast, proportions of targets that lacked mutations decreased with increasingly severe histology. A portion of targets with non-dysplastic and low-grade histology shared a similar ML as those with higher risk and EAC disease. The addition of MSI characterization to ML helped to differentiate the ML between advanced and less advanced histology.

**Conclusions:**

Given that EAC is associated with accumulated genomic instability, high ML in less severe histology may identify BE disease at greater risk of progression to EAC. ML may help to better manage BE in early histological stages and when histology alone provides insufficient information.

## Introduction

Esophageal adenocarcinoma (EAC) exhibits the highest rate of increasing incidence of any solid cancer in the US today, and Barrett's esophagus (BE) represents a precursor of and largest risk factor for EAC [1, 2]. The carcinogenesis of BE has been associated with morphologic changes in esophageal tissue as well as activation of oncogenes and inactivation of tumor suppressor genes [2]. Studies have shown that variable degrees of mutational change take place in the microsatellite regions of tumor suppressor genes at the histological onset of BE [3–7]. The cumulative buildup of various mutations has been closely associated with the different histological grades of BE and EAC [3, 4, 7]. Histological progression to EAC is associated with a relatively poor prognosis, with a 5-year survival rate for regional cancer below 18 % [8]. Consequently, emphasis has been placed upon understanding the risk for progression to EAC of each histological stage of BE such that patients can be appropriately managed with intervention or surveillance.

Accurate histological classification is essential for properly detecting BE at its earliest stages. Guidelines currently define BE as specialized intestinal metaplasia (IM) with goblet cells and grade BE samples with the following progressive histological classifications: IM, indefinite for dysplasia (IND), low-grade dysplasia (LGD), and high-grade dysplasia (HGD) [9]. The most advanced, HGD, has been associated with a greater risk of progression to EAC [10–13].

Both BE and EAC can be readily identified by microscopic examination, but the presence and different grades of dysplasia can be difficult to diagnose due to challenges in discriminating reactive epithelial atypia from true dysplasia [14–18]. The classification of IND is sometimes provided when cellular atypia is observed, but the criteria for the histological diagnosis of dysplasia are not fully met. Poor orientation of the histological sections and presence of inflammatory infiltrate are among the most common factors interfering with the pathologist's ability to differentiate between the presence or absence of dysplasia. Inter-observer variability in the histological classification of BE has been reported by various studies [14–18]. Most variability is linked to LGD [15], yet some variability is also seen in cases of HGD [14], complicating the decision as to how to clinically manage patients.

Non-dysplastic histological features are limited when it comes to determining whether or not a case of BE is likely to progress to cancer or remain stable, as there are no observable non-dysplastic histological microscopic features that can reveal a patient's likelihood of cancer progression. Because of this uncertainty, many choose ablation interventions for low-grade dysplastic and non-dysplastic BE, which has provoked concerns about unnecessary healthcare expenditures [19, 20]. Supplementary diagnostic information that enables better characterization of the risk for less advanced stages of BE disease would be valuable in improving patient care and controlling healthcare costs.

In this study, we aimed to provide additional information to the histological classification of less advanced stages of BE by showing the relationship between BE histology and the presence and extent of genomic instability in multiple study cohorts. Previous investigation has shown that three or more DNA abnormalities in less advanced stages of BE are associated with a higher risk of cancer progression [21]. Two of these abnormalities were loss of heterozygosity (LOH) mutations in microsatellite regions of the *TP53* and *CDKN2A* tumor suppressor genes. Increasing sizes of genetically instable clones with *TP53* and *CDKN2A* LOH have also been associated with increased risk of progression to EAC [22].

We therefore studied the presence and extent (clonality) of genomic instability in advanced and less advanced BE histology in a cross-sectional study of patients from multiple study cohorts. Genomic instability was assessed by mutational load (ML) in histological targets microdissected from BE patient biopsy tissue. ML summarized the presence and extent of LOH next to *TP53* and *CDKN2A* as well as LOH in eight additional genomic loci next to tumor suppressor genes. Microsatellite instability (MSI) around these tumor suppressor genes was also included in the assessment of ML. Our results demonstrate that histology-guided assessment for ML provides an objective measure of the presence and extent of genomic instability. Assessment for ML provides an added dimension to less advanced BE histology that could help to better manage BE patients in early or uncertain histological stages of disease.

## Materials and Methods

### Study Cohort

Standard histological sections (4 μm thick) of formalin-fixed, paraffin-embedded (FFPE) tissue were examined from 415 patients histologically known to have BE. Microdissection of 661 biopsy slides yielded 877 targets in total from three study sites, each with IRB approval of their corresponding study protocol (IRB# 26163, IRB #11-29, IRB # 5658 and IRB# 5629). All patients in the study had previously undergone upper GI endoscopy. Patients without evidence of BE were excluded.

### Histological Classification

Hematoxylin and eosin (H&E)-stained, FFPE histology slides underwent microscopic evaluation for the selection of targets for subsequent microdissection. Each target was histologically classified as follows: normal squamous epithelium, columnar mucosa (COL), and, in order of increasing severity: IM, IND, LGD, HGD, and EAC. BE histological classification began with IM histology. A histological classification was assigned based on pathologist review of tissue. All pathologists were blinded to molecular results. In one study cohort from Norwalk Hospital, three pathologists classified the same microdissected targets. Consensus diagnosis was defined as agreement between at least two pathologists.

### Microdissection

H&E-stained, FFPE slides were used as guides to microdissect tissue (targets) with BE histology from one to three unstained, serial FFPE slides of each patient. Microdissected targets corresponded to distinct foci of tissue with EAC histology, BE histology, COL histology, or normal epithelial histology, as identified by a pathologist. Normal epithelial and COL targets were microdissected from the same FFPE slides as targets with various histological classifications of BE or EAC histology. Microscopic review confirmed the accuracy of all microdissections.

### Detection of LOH and MSI

Detection of LOH and new alleles consistent with MSI were investigated at ten individual genomic loci, using a panel of 22 DNA markers associated with common tumor suppressor genes relevant to BE [3, 23–27]. The presence of MSI at BAT25 and BAT26 loci were also examined in a subset of microdissected targets. LOH and MSI were assessed using polymerase chain reaction (PCR) and quantitative capillary electrophoresis of DNA extracted from each microdissected target, as previous described [24, 25]. DNA markers for the following chromosomal loci comprised the panel (associated genes in parentheses): 1p (*CMM1*, *L-myc*), 3p (*VHL*, *HoGG1*), 5q (*MCC*, *APC*), 9p (*CDKN2A*), 10q (*PTEN*, *MXI1*), 17p (*TP53*), 17q (*NME1*), 18q (*DCC*), 21q (*TFF1 and PSEN2*) and 22q (*NF2*).

Quantitative PCR (qPCR) for housekeeping genes was used to ensure there was sufficient, high quality DNA available for analysis prior to LOH and MSI analysis. The analyses were performed on both BE microdissection samples as well as internal controls (normal appearing squamous and COL), which were all subject to equivalent formalin fixation and histological processing. PCR amplification and subsequent mutational analysis using quantitative capillary electrophoresis methods were then performed on all microdissected samples with adequate qPCR results.

To determine if each LOH analysis was assessable in each patient, the informativeness (heterozygosity) of each LOH marker in normal epithelial from each patient was first examined by quantitative capillary electrophoresis methods. Normal epithelial targets were also used to account for minor differences in the amplification rates of the two-allele lengths during PCR. PCR amplification and subsequent quantitative capillary electrophoresis of DNA from each microdissected target was then performed to assess LOH and MSI.

LOH was called "present" in microdissected targets when there was a degree of allelic imbalance that was equal to or beyond two standard deviations above the average difference in allele peak heights for DNA in normal epithelial microdissection targets. The extent (clonality) of LOH was determined using the ratio of allele peak heights in DNA from microdissected targets, which is proportional to the amount of LOH mutated DNA present in the sample [23–27]. All DNA specimens with LOH were tested in duplicate or triplicate to ensure reproducibility. LOH mutations were considered high clonality when >75 % of the DNA had LOH mutation and low clonality when 50–75 % of the DNA had LOH mutation.

MSI was defined by the presence of additional minor, but reproducible, peaks in the electropherograms after PCR amplification and subsequent quantitative capillary electrophoresis of DNA from each target. The minor peaks did not correspond to either of the two major allele lengths present in normal epithelial control DNA and were not accountable by the presence of shadow band formation during capillary gel electrophoresis. The minor peaks were reproducible through replicate confirmatory PCR amplification and further quantitative capillary electrophoresis testing.

### Mutational Load

**Fig. 1:**
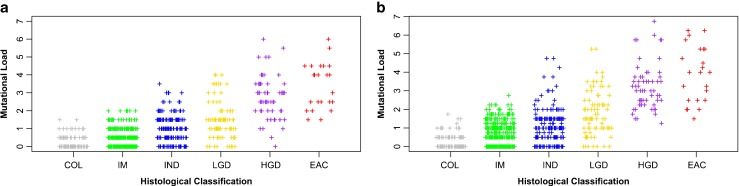
Correlation of mutational load (*ML*) to histological classification of microdissected targets **a** based on the presence and extent of low and high clonality LOH mutations only (correlation coefficient = 0.68, *p* < 0.0001) and **b** based on the presence and extent of low and high clonality LOH mutations as well as MSI (correlation coefficient = 0.69, *p* < 0.0001). *COL* columnar mucosa, *IM* intestinal metaplasia, I*ND* indefinite for dysplasia, *LGD* low-grade dysplasia, *HGD* high-grade dysplasia, *EAC* esophageal adenocarcinoma

ML measured the presence and clonality of LOH mutations and the presence of MSI at each genomic locus examined. The presence and clonality of LOH mutation at each genomic locus was determined for each microdissected target. Low clonality LOH was defined as 50–75 % of the DNA containing LOH, and high clonality LOH was defined as >75 % of the DNA containing LOH. All LOH mutations at a given genomic loci were assigned a numerical value based on their low or high clonality. A proportional value of 0.5 was assigned for low clonality mutations and 1 for high clonality mutations using proportional odds logistic regression (POLR), as previously described [3]. The presence of MSI was also assigned a proportional value using POLR. The proportional value of MSI present at a single genomic locus was 0.75. The proportional value of each additional MSI present, beyond one locus, was 0.5. These numerical values for low clonality and high clonality LOH mutations (Fig. 1a) as well as MSI mutations (Fig. 1b) were added together for all loci containing LOH and/or MSI in a microdissected target. The resulting cumulative value was defined as the ML for that microdissected target.

Polyserial correlation coefficient was used to examine the correlation between histological class and ML of microdissected targets when ML was assessed using only LOH mutations (Fig. 1a) or both LOH and MSI mutations (Fig. 1b). An analysis of variance (ANOVA) model was used to examine the difference in ML between advanced (HGD, EAC) and less advanced (IM, IND, LGD) histological classifications when ML included only LOH analysis as compared to when it included both LOH and MSI analysis. ANOVA was performed with an interaction term between the two methods of ML assessment (LOH only, LOH and MSI) and the two categories of histological classifications of BE (advanced histology, less advanced histology).

Three levels of ML were defined using the distribution of ML present in the population of targets with IM histology. (1) "No ML" contained microdissected targets that lacked mutations. (2) "Low ML" contained targets that had mutations, but the level of ML in this category was below the top 5th percentile of IM targets that had the highest ML. (3) "High ML" contained microdissected targets with an ML similar to those targets in the top 5th percentile of IM targets with the highest ML. These levels of ML were applied to all histological classifications.

## Results

### The Presence of LOH and MSI in Histological Classifications

Mutational analysis was performed on 877 microdissected targets corresponding to 661 esophageal biopsy slides from 415 patients. The average number of low clonality and high clonality LOH mutations and MSI mutations in microdissected targets generally increased with increasingly severe histology (Table 1). MSI mutations occurred less often than LOH mutations, but generally increased with increasingly severe histological classifications. LOH mutations were also most abundant in targets with dysplastic histology and occurred on average more often than MSI in HGD and EAC. Low clonality LOH and MSI mutations were detected in similar or higher abundance than high clonality LOH mutations in less severe histological classifications (IM, IND, LGD), suggesting these mutations may occur prior to the appearance of more advanced histological stages of BE.

**Table 1:** Average number of mutations detected in microdissected targets with each histology

Histological classification	Total micro. targets tested	Average number of mutated loci detected per micro. target	Average number of low/high clonality LOH and MSI mutations detected per micro. target			Average mutational load (ML)
			Low	High	MSI	
COL	99	0.5	0.5	0.0	0.0	0.3
IM	427	1.1	0.7	0.1	0.3	0.7
IND	182	1.9	1.4	0.3	0.2	1.1
LGD	85	2.7	1.7	0.6	0.5	1.8
HGD	61	4.3	2.2	1.6	0.8	3.2
EAC	23	5.9	4.3	1.3	0.8	3.9

*COL* columnar mucosa, *IM* intestinal metaplasia, *IND* indefinite for dysplasia, *LGD* low-grade dysplasia, *HGD* high-grade dysplasia, *EAC* esophageal adenocarcinoma, *Micro.* microdissected

Mutations were observed across the entire panel of genomic loci examined. Table 2 describes the percentages of microdissected targets mutated at each of the ten genomic loci for each histological classification. The percentages of LOH and MSI mutated targets generally increased with more advanced histological classifications, from COL to HGD. Importantly, LOH and MSI mutations at all loci were detected with less advanced stages of BE histology (IM, IND, LGD) but were found more frequently with more advanced stages of BE histology (HGD, EAC). In less advanced BE histology (IM, IND), the most frequently LOH mutated loci included 9p (*CDKN2A*), 10q (*PTEN, MXI1*), and 17p (*TP53*), 17q (*NME1*), which is consistent with previous studies [3, 21, 22, 28]. MSI mutations at nearly all loci occurred at a similar frequency in less advanced stages of BE histology. In targets with COL, limited MSI and LOH mutations were observed across the panel of loci examined.

**Table 2:** The percent microdissected targets with mutations each at each genomic loci by histological classification

Mutation	Loci	Tumor suppressor genes (TSG)	The percent of microdissected targets with LOH and MSI mutations at each loci in each Histological Classification					
			% COL *N* = 99	% IM *N* = 427	% IND *N* = 182	% LGD *N* = 85	% HGD *N* = 61	% EAC *N* = 23
MSI	1p	*CMM1, LMYC*	1	–	2	1	7	9
	3p	*VHL, OGG1*	–	7	3	11	13	3
	5q	*MCC, APC*	–	3	3	7	–	4
	9p	*CDKN2A*	2	2	–	1	13	–
	10q	*PTEN, MXI1*	–	1	3	2	10	9
	17p	*TP53*	–	4	2	7	7	9
	17q	*NME1*	–	2	3	14	11	4
	18q	*DCC*	–	5	1	1	10	4
	21q	*TFF1, PSEN2*	–	3	–	1	–	–
	22q	*NF2*	–	2	–	4	5	4
LOH	1p	*CMM1, LMYC*	1	5	14	19	34	43
	3p	*VHL, OGG1*	2	5	18	27	41	61
	5q	*MCC, APC*	11	8	18	20	34	61
	9p	*CDKN2A*	7	32	36	45	72	61
	10q	*PTEN, MXI1*	10	7	22	8	15	43
	17p	*TP53*	11	11	29	49	87	83
	17q	*NME1*	2	7	22	26	21	65
	18q	*DCC*	–	1	1	11	38	78
	21q	*TFF1, PSEN2*	5	5	12	24	21	13
	22q	*NF2*	–	2	1	5	15	48

*COL* columnar mucosa, *IM* intestinal metaplasia, *IND* indefinite for dysplasia, *LGD* low-grade dysplasia, *HGD* high-grade dysplasia, *EAC* esophageal adenocarcinoma, – 0 % targets mutated

### Pathologist Variability Amongst Histological Classifications

To understand which histology was most reliably classified for each microdissected target, the agreement amongst three pathologists was examined in one study cohort (Table 3). Consistent with previous reports, most disagreement was linked to IND and LGD histology, where in the majority of microdissected targets at least one pathologist disagreed with another (100 % IND and 88 % LGD) [15]. By contrast, all three pathologists agreed on the histological classification of IM in the majority of microdissected targets (69 %). In 50 % of microdissected targets all pathologists agreed in classifying HGD, while in the other 50 % of targets one disagreed, consistent with previously published studies describing variability amongst HGD calls [14].

**Table 3:** Frequency of pathologist agreement on histological classification in one study cohort

	% All pathologists agree	% One pathologist disagrees	% All pathologists disagree
IM (*N* = 115)	69	30	1
IND (*N* = 19)	0	63	37
LGD (*N* = 8)	13	88	0
HGD (*N* = 4)	50	50	0

*IM* intestinal metaplasia, *IND* indefinite for dysplasia, *LGD* low-grade dysplasia, *HGD* high-grade dysplasia

### Assessment of Genomic Instability

The presence and extent (clonality) of genomic instability in each microdissected target was assessed by ML. The ML of a target was calculated based on the presence and clonality of LOH mutations as well as the presence of MSI in DNA from each histological target. In this system, numerical values were determined by POLR as follows: 0.5 for low clonality LOH mutations (50–75 % of DNA had LOH), 0.75 for the first MSI, 0.5 for each additional MSI, and 1 for high clonality LOH mutations (>75 % of DNA had LOH). These numerical values for low clonality LOH, MSI, and high clonality LOH mutations were added together for all loci in a microdissected target. The resulting cumulative value was defined as the mutation load for that microdissected target. Consistent with a recent publication in a smaller dataset, the ML of microdissected targets positively correlated with increasingly severe histology (Fig. 1) [3]. However, ML in this past publication was only assessed using the presence of low and high clonality LOH mutations. The correlation of increasingly severe histology to ML based on the presence of low and high clonality mutations alone is shown in Fig. 1a (correlation coefficient = 0.68, *p* < 0.0001). When a weighted value for MSI mutations was included in the assessment of ML (Fig. 1b), the positive correlation between ML and increasingly severe histology was slightly improved (correlation coefficient = 0.69, *p* < 0.0001). Importantly, the addition of MSI to the assessment of ML helped to better discriminate the difference in ML between less advanced (IM, IND, LGD) and more advanced (HGD, EAC) histological classifications of BE (Fig. 1a vs. Fig. 1b). The average difference in ML between less advanced histology (IM, IND, LGD) and more advance histology (HGD, EAC) was statistically higher when both LOH and MSI was considered as compared to only LOH (2.54 vs. 2.21, *p* = 0.02).

Using the distribution of ML present in the population of targets with IM histology, three levels of ML were established with respect to histological classifications when both LOH and MSI were included in the assessment of ML (Table 4). The first level contained microdissected targets that lacked mutations and, as such, had "no ML". The second level ("low ML") contained targets with an ML found in the majority of IM microdissected targets. Low ML targets had mutations but were below the top 5th percentile of IM targets with the highest ML. The third level ("high ML") contained microdissected targets with an ML similar to those targets in the top 5th percentile of IM targets with the highest ML.

**Table 4:** Percentage of targets with each level of mutational load (ML) by histological classification

Histology of microdissected targets	Mutational load (ML)		
	% No ML	% Low ML	% High ML
COL (*N* = 99)	61	38	1
IM (*N* = 427)	30	62	8
IND (*N* = 182)	18	66	16
LGD (*N* = 85)	8	46	46
HGD (*N* = 61)	0	5	95
EAC (*N* = 23)	0	4	96

*COL* columnar mucosa, *IM* intestinal metaplasia, *IND* indefinite for dysplasia, *LGD* low-grade dysplasia, *HGD* high-grade dysplasia, *EAC* esophageal adenocarcinoma

Table 4 summarizes the proportion of microdissected targets for each level of ML in each histological class. The majority of microdissected histological targets composed of COL had no Detectable ML (Table 4, no ML). Of the proportion of non-BE targets (COL) that had mutations, all but one were low ML. Of the IND microdissected targets 18 % had no ML, while the remaining proportion of IND targets had mutations, including 66 % that had low ML and 16 % that had high ML. Most microdissected targets histologically classified as LGD had mutations with the majority of targets falling into the low ML (46 %) or high ML (46 %) levels. Nearly all HGD (95 %) and EAC (96 %) histological targets had high ML. Comparatively, only 8 % of IM and 16 % of IND microdissected targets were characterized as having a high ML.

## Discussion

Current risk stratification of BE patients for EAC relies on histological classification and grade of dysplasia. However, histological classification alone cannot sufficiently assess the risk of patients with variable or non-dysplastic BE histology [2, 29]. Progression of BE patients to EAC is associated with accumulated genomic instability [4, 30]. We therefore examined differences in the presence and extent of genomic instability in advanced and less advanced stages of BE histology. Genomic instability was assessed by ML, accounting for the presence and clonality of LOH as well as the presence of MSI in ten genomic loci around tumor suppressor genes associated with EAC (Tables 1 and 2). Our results demonstrate that histology-guided assessment for ML provided an objective measure of genomic instability amongst BE histological classifications.

Consistent with previous work, increasing ML correlated with increasingly severe BE histology (Table 1, Fig. 1) [3]. The addition of MSI characterization at each of the ten genomic loci to the assessment of ML slightly increased this correlation (Fig. 1a and b). Studies have published conflicting results regarding the association of MSI and EAC [31, 32]. We therefore examined MSI at BAT25 and BAT26 in a subset of microdissected targets (*N* = 71). In this subset, MSI at BAT25 or BAT26 was detected in 4/43 microdissected targets histologically classified as HGD or EAC (data not shown). However, MSI in these markers was not detected in less severe BE histology (IM, IND, LGD), suggesting that MSI at these loci had little predictive value concerning disease progression in this subset. By contrast, the appearance of new alleles indicative of MSI was consistently observed in the microsatellite repeats of the ten genomic loci in which LOH was also examined, both in less advanced (IM, IND, LGD) and more advanced histological classifications (HGD, EAC) (Table 2). To our knowledge, this is the first report of MSI related to BE in these microsatellites, which are composed of tetranucleotide repeats. The novel detection of MSI at these ten loci in less severe histological classifications of BE (IM, IND, LGD) could be a result of MSI produced by mechanisms different than those associated with BAT25 and BAT26 mononucleotide repeats, which involve disruption of DNA repair (*hMLH1/hMSH2*) functions. In general, MSI at these ten genomic loci slightly increased with increasingly severe histology (Table 2). More importantly, the addition of MSI to the assessment of ML helped to better stratify the difference in ML between less advanced (IM, IND, LGD) and more advanced (HGD, EAC) histological classifications of BE (Fig. 1).

We defined three levels of ML (no, low, and high ML), when MSI mutations and low and high clonality LOH mutations were assessed. These three levels were determined using the heterogeneous distribution of ML present in the population of targets with IM histology (*N* = 427). The levels were then applied to each histological classification to determine the proportion of targets captured in each level (Table 4). The three levels were stratified using the distribution of ML in IM targets, because IM histology was the most reliably classified histology amongst all three pathologist in the subset of microdissected targets analyzed here, which has also been observed by others (Table 3) [14, 17].

High, low, and no levels of ML captured relatively similar proportions of microdissected targets with each histology as compared to that of a past single cohort study [3]. Importantly, microdissected targets with HGD (95 %) or EAC (96 %) consistently had high ML (Table 4) and about half of LGD (46 %) histological targets had high ML in both studies. Although the presence of HGD and confirmed LGD are risk factors for progression to EAC, lack of pathologist agreement concerning LGD and IND histological classifications, as observed here and in other studies, suggests a need for additional, more objective clinical information when such diagnoses are encountered (Table 3) [11, 13, 29]. ML can provide information about genomic instability for these inconsistent histological classifications of BE.

Roughly half of the histological targets (44 %) with high ML had high risk or severe histological classifications (HGD, EAC), while the other half (56 %) had less severe histology (COL, IM, IND, LGD). Importantly, a proportion of non-dysplastic BE targets with IM (8 % high ML) and IND (16 % high ML) histology had a similar ML as those with HGD and EAC histology (Table 4). Therefore, this portion of targets had molecular changes that are similar to those seen with high risk and severe disease (HGD, EAC), which is consistent with that observed in previous work concerning ML [3]. High ML in less severe histological classifications of BE may be indicative of imminent morphological changes that have yet to become visible by histology. A relatively high load of molecular changes, which occurs in a fraction of microdissected targets with less advanced histology, may help to more objectively identify targets that are more likely to develop into advanced BE histology (HGD or EAC).

By contrast, mutations were not always present in microdissected targets with less severe histology (COL, IM, IND, LGD). The proportion of microdissected targets that lacked mutations (no ML) decreased with increasingly severe histology (Table 4), consistent with previous work describing ML [3]. Importantly, lack of mutations (no ML) was not detected in any microdissected targets with HGD or EAC histology. Because the loci examined in this study are a survey of genomic sites relevant to Barrett's associated EAC, the absence of clonally expanded LOH mutations and MSI mutations next to these ten relevant genomic loci provides strong evidence that the histological targets examined did not have extensive genomic instability related to BE. Therefore, the lack of ML (no ML) in a portion of BE targets with less severe histology is likely indicative of benign biological processes. In addition, low levels of mutations (low ML) were present in COL, suggesting that the presence of a few mutations alone is not necessarily indicative of higher risk histological disease.

Our study is consistent with others describing genomic instability around *TP53* and *CDKN2A* tumor suppressor genes, which has been associated with greater risk of BE histological progression to EAC [21, 22, 28]. Similar to these studies, LOH in the microsatellite regions of *TP53* and *CDKN2A* tumor suppressor genes were included in our panel. LOH mutations in these loci were found most frequently in microdissected targets with histological dysplasia (Table 2). However, consistent with previous work, many additional genomic loci also had LOH in dysplastic targets and targets with EAC, suggesting that all loci are relevant to progressive Barrett's disease [3]. Furthermore, many genomic loci had LOH mutations in non-dysplastic targets (IM, IND) in comparable frequency to *CDKN2A* and *TP53* associated mutations, suggesting that other genomic loci beyond those related to *TP53* and *CDKN2A* are present at even early stages of BE. Similarly, MSI mutations at these ten genomic loci occurred more often in dysplastic microdissected targets but were also present in non-dysplastic targets across nearly all loci.

While the strengths of this study have demonstrated that ML can provide an objective measure of the presence and extent of genomic instability amongst BE histological classifications, there are study limitations to consider. Limitations in this study include those inherent to studies correlating information to histological diagnoses. Due to variability in pathologist diagnoses, there is unavoidable variability in the histological classification used as the reference standard when correlating molecular results. However, when consensus histological classification was compared to ML in a subset of microdissected targets, the correlation between histological diagnosis and ML was still observed (data not shown), although only limited dysplastic histological targets were available for multiple pathologist review in this subset. Moreover, this study is cross-sectional. Further studies are required, and ongoing, to determine how accurately levels of ML described herein can differentiate patients who have less severe BE histology (IM, IND, LGD) and will progress from those who will not progress over time.

The results of this study support the use of ML assessments in conjunction with histological classification of BE to better manage patients. ML assesses the presence and extent of genomic instability, which has been associated with increased risk of progression to EAC [4, 30]. A portion of microdissected targets with non-dysplastic histological classifications shared a similar ML as those with higher risk disease (HGD) and even EAC. Given that EAC is associated with accumulated genomic instability, high ML in less severe histological diagnoses may be an indication of BE disease at higher risk of progression to EAC. Therefore, ML may help to better manage BE patients in early stages of disease and when histological diagnosis provides insufficient information. Incorporating assessments for ML in patient management may help to reduce health economic costs and increase patient quality of life by limiting unnecessary clinical interventions and frequent surveillance in patients destined for non-malignant disease and by allowing earlier and less morbid intervention in patients destined for malignancy.
